# Prevalence of pregnancy induced hypertension and pregnancy outcomes among women seeking maternity services in Harare, Zimbabwe

**DOI:** 10.1186/s12872-015-0110-5

**Published:** 2015-10-02

**Authors:** Monica Muti, Mufuta Tshimanga, Gombe T. Notion, Donewell Bangure, Prosper Chonzi

**Affiliations:** Department of Community Medicine, University of Zimbabwe, P.O. Box A178, Avondale, Harare, Zimbabwe; Harare City Health Department, Harare, Zimbabwe

**Keywords:** Pregnancy induced hypertension, Pregnancy outcome, Harare City

## Abstract

**Background:**

Pregnancy induced hypertension (PIH) is one of the most common causes of both maternal and neonatal morbidity, affecting about 5 – 8 % of pregnant women. It is associated with adverse pregnancy outcomes as well as maternal morbidity and mortality. Harare City experienced an increase in referrals due to PIH to central hospitals from 2009 to 2011. We conducted a study to determine the prevalence of PIH and pregnancy outcomes among women with PIH.

**Methods:**

An analytic cross sectional study was conducted. Interviewer administered questionnaires were used to capture demographic data, obstetric history and knowledge on PIH management. Records were reviewed for pregnancy outcomes while key informants were also interviewed on patient management.

**Results:**

PIH prevalence was 19.4 %. Women with PIH were three times more likely to deliver a low birth weight baby (OR 3.00, *p* = 0.0115), 4.3 times more likely to have still birth (OR 4.34, *p* = 0.0517) and four times more likely to have a baby with low Apgar score at 5 minutes (OR 4.47, *p* = 0.0155) compared to women without PIH. There was no statistically significant difference in delivery before 37 weeks gestation between women with PIH and those without (OR 1.70, *p* = 0.1251). 12,5 % of the women delivered by caesarean section. Methyldopa was the drug of choice for management of PIH. Less than half of the health workers had sufficient knowledge on definition or management of PIH. Delay in seeking care and shortage of resources were the major reported challenges in the proper management of PIH.

**Conclusion:**

PIH prevalence was high. Women with PIH were at higher risk of adverse pregnancy outcomes than those without. Poor knowledge of management of PIH and inadequate resources are a threat to the proper management of PIH. This underscores the need for increased human resources and capacity building as well as resource mobilisation for proper management of pregnant women. Urinalysis must be routinely done for all pregnant women regardless of their blood pressure.

## Background

Worldwide, 10 % of all pregnancies are complicated by hypertension, with pre-eclampsia and eclampsia being the major causes of maternal and prenatal morbidity and mortality [1]. It is also estimated that pregnancy induced hypertension (PIH), one of the hypertensive disorders of pregnancy, affects about 5 – 8 % of all pregnant women worldwide [2]. Pregnancy induced hypertension (PIH) is defined as BP ≥ 140/90 mmHg, taken after a period of rest on two occasions or ≥160/110 mmHg on one occasion in a previously normotensive woman [3].

Pre-eclampsia affects 5-7 % of all pregnancies. It is broadly defined by hypertension and proteinuria [4]. Eclampsia includes pre-eclampsia with the presence of convulsions not attributable to other neurologic disease.

PIH is a major pregnancy complication associated with premature delivery, intra-uterine growth retardation (IUGR), abruptio placentae, and intra-uterine death, as well as maternal morbidity and mortality [5, 6]. It is estimated that 9.1 % of maternal deaths in Africa are due to hypertensive disorders of pregnancy [1]. In Zimbabwe, neonatal causes of under five mortality which comprise preterm birth complications, birth asphyxia and neonatal sepsis contribute to 29 % of the deaths. 39 % of neonatal deaths are caused by preterm birth complications [7]. The Zimbabwe Maternal and Perinatal mortality study of 2007 found PIH to be among the top five causes of maternal mortality and the third highest reason for referral in labour [8].

Tachiwenyika et al. also found that PIH was associated with an increased risk of perinatal mortality [9]. On the contrary, Hauth et al. found in their study that fetal and neonatal mortality were similar in women with hypertension and those without. However, selected maternal and newborn morbidities such as increased cesarean deliveries, abruptio placentae, and acute renal dysfunction, respiratory distress syndrome, ventilatory support, and fetal growth restriction were significantly greater in women with hypertension [10].

PIH has also been found to be a risk factor for low birth weight. Rahman et al found PIH to be an independent risk factor for low birth weight. Women who delivered low birth weight babies were 5 times more likely to have had pregnancy-induced hypertension [11].

A notable increase in referrals of pregnant women due to PIH from the Harare local authority health facilities to central hospitals was noted in Harare (from 20.7 % in 2009 to 44 % in 2011). The concern over the increase in PIH and the possible consequences necessitated an investigation into the prevalence and pregnancy outcomes among women with this condition, seeking maternity services in Harare. Prior to this study, no investigation had been done by the City Health Department to establish the characteristics of the women affected or how they were being managed. It was also not clear whether the rise in referrals due to PIH was a reflection of high prevalence of PIH or case management at primary care level.

This study was conducted to establish the prevalence of pregnancy-induced hypertension (PIH) as well as foetal and maternal outcomes among women seeking maternity services in Harare. Specifically we wanted to determine the characteristics of women with PIH in Harare, the prevalence of PIH in women seeking ANC services in Harare, the maternal outcomes for women with PIH, the foetal outcomes for women with PIH and assess case management of pregnant women with PIH.

Results of the study will be used to inform reproductive health programming in Harare, future management of pregnancy induced hypertension as well as areas for further investigation.

## Methods

**Fig. 1:**
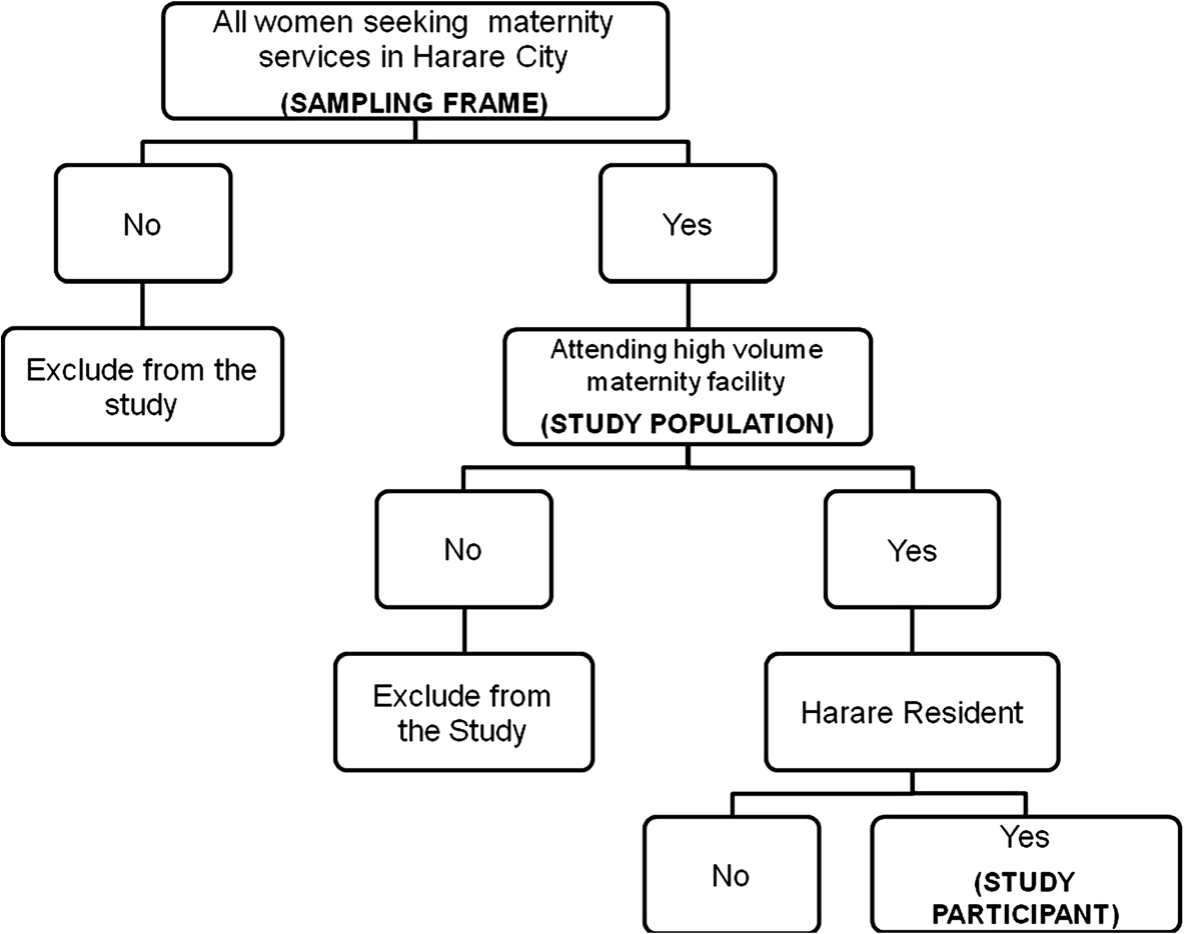
Consort Diagram for PIH Study Participants: Harare, Zimbabwe, 2012

An analytic cross-sectional study was conducted at Harare Central Hospital and 6 out of the 12 Harare City maternity units. The Central Hospital was purposely selected while the other maternity units were selected based on their volumes of deliveries. The sites with the highest numbers of deliveries (high volume sites) were included in the study. All women delivering at Harare Central Hospital and the selected six maternity units in September 2012 were the study, participants. Those who were very ill and unable to participate in interviews as well as women delivering at the study sites who were not Harare residents were excluded from the study as shown in Fig. 1. The residence was ascertained by asking the usual place of residence for the prospective study participant.

### Data collection

Two data collectors and a lead researcher conducted the data collection. Interviewer-administered questionnaires were used to collect demographic data and obstetric history from women soon after delivery.

Key Informant Interview Guides were used to collect information on PIH management from key informants. Key informants included the Reproductive Health officer, The Assistant Director of Health-Nursing and the nurses in charge of the maternity units where data was being collected.

Nurses found on duty were interviewed using interviewer-administered questionnaires to establish how pregnant women with PIH were managed at the health facility level.

Records and delivery registers were reviewed for patient management and pregnancy outcomes. Maternal and foetal outcomes were assessed using delivery registers and individual maternity booklets. Individual maternity booklets were also reviewed to establish how they were managed during labour and delivery as well as post partum.

For the purposes of this study, the following definitions were used:

Mild PIH is classified as blood pressure BP ≥ 140/90 mmHg and severe PIH is blood pressure ≥ 160/110 mmHg. Clinical manifestation usually begins after 20 weeks of gestation and resolves by the 6^th^ week post partum.

Apgar Score was defined as a ten point scale used to determine the prognosis of a new born infant and was obtained from individual maternity booklets of the mothers

Low birth weight was defined as a birth weight of below 2.5 kg

Preterm birth was defined as birth below 37 weeks gestation

### Sample size calculation and sampling procedures

The following formula for calculating sample size for a single proportion was used to calculate the sample size for women to be enrolled into the study:

$$ \mathrm{n} = {\mathrm{z}}^2/{\mathrm{d}}^2 \times \mathrm{p}\mathrm{q} $$

Where z = risk of Type I error

d = absolute precision

p = expected prevalence

q = 1-p

Using 95 % confidence interval, 80 % power and estimated precision of 5 % and assuming the prevalence of PIH among pregnant women to be 24.9 % (based on a study by Hauth et al in USA on pregnancy outcomes among healthy nulliparas who developed hypertension), the estimated sample size for women to be enrolled into the study was:

$$ \begin{array}{l}\mathrm{n} = 1.962/0.052 \times \left(0.249\times 0.751\right)\\ {}\kern0.48em =287\end{array} $$

The six study sites under Harare City Council jurisdiction were selected based on their volumes of deliveries, with the sites with the highest volumes of deliveries being included into the study. The following were the expected number of participants per participating health facility: Harare Central Hospital 109, Edith Opermann Maternity Clinic 50, Rutsanana Maternity Unit 30, Mabvuku Polyclinic 29, Budiriro Polyclinic 25, Kuwadzana Polyclinic 24 and Rujeko Polyclinic 20 participants. The quality and type of services offered at all the twelve maternity units is similar, with the Central Hospital being the only one that offers unique services such as cesarean section and management of pre-eclampsia or eclampsia. The number of participating women at each study site was proportionally assigned according to the volume of deliveries. The delivery register was reviewed to establish the number of new deliveries on the day of the study. All women meeting the inclusion criteria were interviewed until the required sample size for the site was reached.

All nurses found on duty were interviewed.

### Data analysis

Quantitative data was captured and analyzed using the Epi-info statistical package to generate frequencies, means and odds ratios. Microsoft Excel was used to generate graphs.

### Permission to proceed and ethical considerations

Permission was sought and obtained from the District Medical Officers, Nurses in Charge of Health facilities, the Chief Executive Officer of Harare Central Hospital and the Harare Central Hospital Ethical Review Board and the Matron in charge of Harare Maternity Hospital.

Written and informed consent was sought and obtained from all study participants. Confidentiality was assured and maintained throughout the study. Names of participants were not captured on questionnaires.

Necessary measures were taken to avoid disrupting the daily patient care activities. Nurses were only interviewed at times when they were not caring for patients.

## Results

During the study period, there was a total of 2375 deliveries in Harare. None of the women at the study sites was found to be too ill to respond to the questionnaire. Twenty-five health workers and 289 women were interviewed post partum. Fifty-six (56) of the post partum women were found to have PIH thus the prevalence of PIH was 56/289 × 100 = 19.4 %. Those with PIH were older than those without PIH (median age 29 and 25 respectively, *p* = 0.0009) (Table 1). The prevalence of pre-eclampsia was 1.7 % and the prevalence of eclampsia was 0.3 %.

**Table 1:** Demographic characteristics of study participants: Harare, Zimbabwe, September 2012

Variable		With PIH *N* = 56 (n,%)	Without PIH *N* = 233 (n.%)
Occupation	Formally employed	2 (3.6)	21 (9.0)
	Informally employed	2 (3.6)	7 (3.0)
	Self Employed	10 (17.9)	27 (11.6)
	Student	1 (1.8)	2 (0.9)
	Unemployed/housewife	41 (73.2)	176 (75.5)
Education level	Never been to school	0 (0)	1 (0.4)
	Primary	3 (5.4)	25 (10.7)
	Secondary	50 (89.3)	198 (85.0)
	Tertiary	3 (5.4)	9 (3.9)
Marital status	Divorced	0 (0)	3 (1.3)
	Married	55 (98.2)	228 (97.9)
	Never married	1 (1.8)	2 (0.9)
Median age in years		29	25

**Fig. 2:**
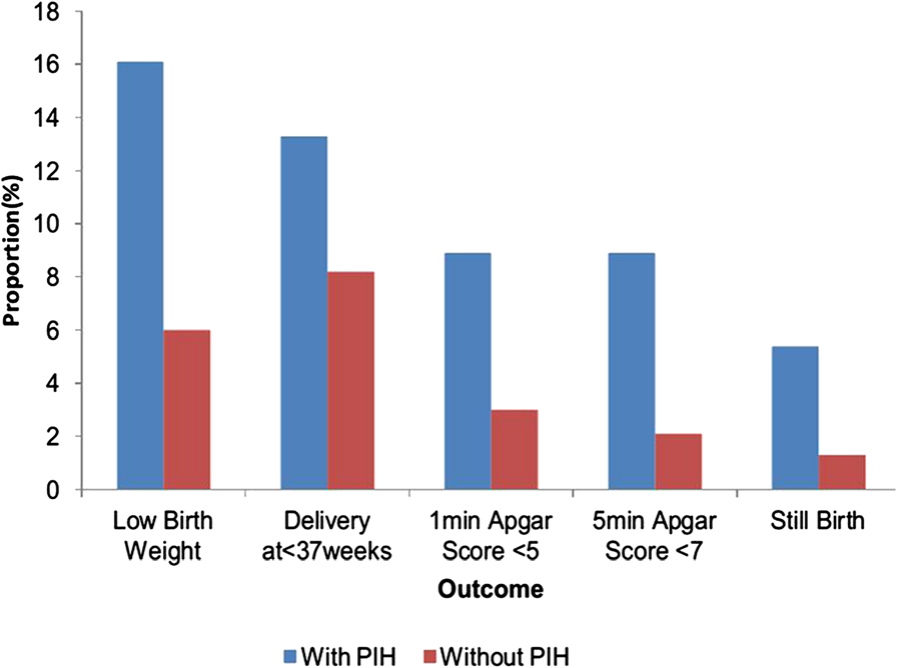
Foetal outcomes of study participants, Harare, Zimbabwe, 2012

Of the babies born to mothers with PIH, nine (16.1 %) had low birth weight compared to 14 (6 %) among those born to mothers without PIH. Of those women who had PIH, 8 (14.3 %) delivered their babies before 37 weeks gestation compared to 19(8.2 %) among women without PIH (Fig. 2).

**Fig. 3:**
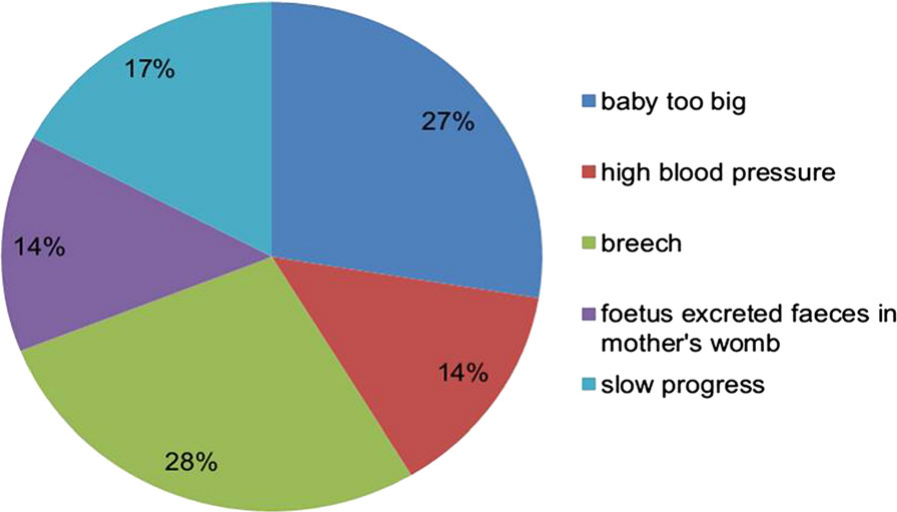
Reasons for caesarean section among study participants: Harare, Zimbabwe, 2012

Thirty-six respondents (12.46 %) delivered by caesarean section. Three (1.04 %) had an assisted delivery. The major reasons for caesarean section delivery were macrosomia, breech presentation and slow progress **(**Fig. 3).

**Fig. 4:**
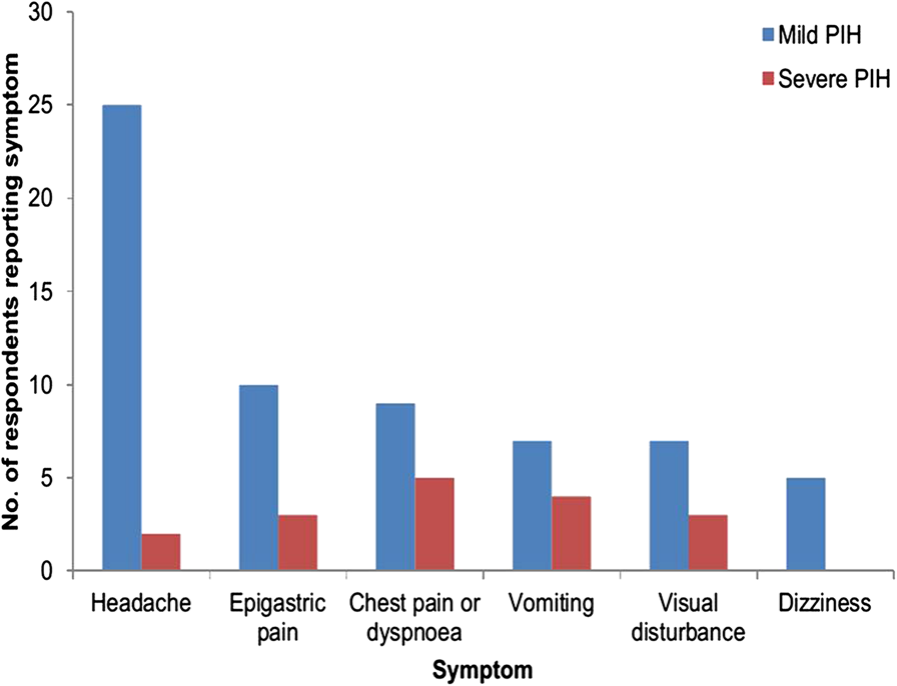
Common symptoms experienced by PIH study participants: Harare, September 2012

Among the women with mild PIH, 5 (10.4 %) developed pre-eclampsia while one (2.1 %) developed eclampsia. None of those with severe PIH developed eclampsia. The most common symptom experienced was a headache, with 25 (52.1 %) of those who had mild PIH experiencing a headache and five out of the eight women with severe PIH reporting having experienced a headache. Other symptoms experienced were epigastric pain (23.2 %), chest pain or dyspnoea (21.4 %), visual disturbance (19.6 %), vomiting (16.1 %) and dizziness (8.9 %) (Fig. 4). Oedema was present in 22 (39.3 %) of the respondents who experienced PIH.

Methyldopa was the drug of choice for management of PIH. It was administered to 20 (45.5 %) of those who had mild PIH and 3 out the 4 with severe PIH. Methyldopa and nifedipine were given in combination to four (9.1 %) women with mild PIH and one with severe PIH. Hydralazine was given to one woman with mild PIH (Table 2).

**Table 2:** Management of women with PIH in Harare, Zimbabwe, September 2012

Medication	Mild PIH (BP140/90mmHg) *N* = 41 (n,%)	Severe PIH (BP ≥ 160/110mmHg) *N* = 15 (n,%)
No Medication/Bed rest	16 (39.0)	3 (20.0)
Methyl Dopa	19 (46.3)	9 (60.0)
Nifedipine	2 (4.9)	1 (6.7)
Methyldopa + nifedipine	4 (9.8)	1 (6.7)
Hydralazine	0	1 (6.7)

Women aged above 35 were almost five times more likely to suffer from PIH compared to younger women (*p* = 0.0024). Nullips were less likely to have PIH and the association was statistically significant (OR 0.31, *p* = 0.0454. Those with a previous history of PIH were 6.85 times more likely to suffer from PIH with the current pregnancy compared to those with no previous history of PIH (*p* = 0.0000).

Current PIH was associated with poor foetal outcomes. Women with PIH were three times more likely to deliver a low birth weight baby (OR 3.00, *p* = 0.0115), 4.3 times more likely to have still birth (OR 4.34, *p* = 0.0517) and four times more likely to have a baby with low Apgar score at 5minutes (OR 4.47, *p* = 0.0155) compared to women without PIH. There was however no statistically significant difference in delivery before 37 weeks gestation between women with PIH and those without (OR 1.70, *p* = 0.1251) (Table 3).

**Table 3:** Bivariate analysis of foetal outcomes of study participants: Harare, Zimbabwe, 2012

Outcome		With PIH	Without PIH	OR	*p*-Value
		*N* = 56 (n, %)	*N* = 233 (n, %)		
Foetal Birth weight	Low BW (<2500g)	9 (16.1)	14 (6.0)	3.00	0.0115
	Normal BW	47 (83.9)	219 (94.0)		
Still birth	Yes	3 (5.4)	3 (1.3)	4.34	0.0517
	No	53 (94.6)	230 (98.7)		
Delivery at < 37 weeks	Yes	8 (14.3)	19 (8.2)	1.70	0.1251
	No	48 (85.7)	214 (91.8)		
1minute apgar score <5	Yes	5 (8.9)	7 (3.0)	3.17	0.0376
	No	51 (91.1)	226 (97.0)		
5 minute apgar score <7	Yes	5 (8.9)	5 (2.1)	4.47	0.0155
	No	51 (91.1)	228 (97.9)		

### Results of interviews with health workers and key informants

Less than half of the health workers had sufficient knowledge on the definition or management of PIH. Eleven of the 25 health workers interviewed defined PIH as blood pressure of 130/90 mmHg. Only seven mentioned that it is from 20 weeks gestation and one mentioned that it occurs up to 6weeks post delivery. Only seven respondents could correctly state how PIH is managed according to the reproductive health guidelines.

Conditions mentioned for referral of women with PIH include blood pressure equal to or above 130/90 with proteinuria and oedema or if in labour, failure to respond to treatment and a diastolic pressure of greater than or equal to 110mmHg during ANC or a diastolic pressure of greater than or equal to 110 in labour. Key informants thought that the reasons for an increase in admissions due to PIH could be associated with an increased awareness of the condition by nurses thus improved diagnosis, improved health seeking behavior of pregnant women and the increase in bookings with consequent increase in detection of the condition as well as the desire of primary level midwives to save lives of both the mother and baby.

Focused ANC, educating women on the importance of regular blood pressure checks and early booking were mentioned as potential ways of reducing the burden of PIH. Key informants thought nothing can be done about the growing burden of referrals due to PIH as they thought it’s not about referrals but about saving lives. To quote “*Midwives should not take risks. Referrals reflect alertness. Better technology and obstetricians are found at tertiary level.”*

Various challenges were mentioned in the management of PIH. Delay in seeking care was reported to result in the use of more resources. Seventeen respondents reported that shortage of resources challenged proper management of PIH. Functional urine sticks, BP machines and human resources for close monitoring of clients were reported to be in short supply. Urinalysis was conducted only for those with elevated blood pressure due to the shortage of functional urine sticks. Seventeen respondents mentioned transport as a challenge in the referral of clients. Delays in ambulance response last up to four hours and clients often do not have money for alternative transport. Other challenges mentioned include clients refusing to be referred to tertiary level, unbooked mothers who cannot afford maternity booking fees, poor drug compliance and too many patients thus compromising the quality of patient care.

## Discussion

In our study, previous history of PIH was associated with increased risk of PIH in the current pregnancy. According to Cande et al, the occurrence of PIH in one pregnancy is a strong predictor of recurrence in the next pregnancy and recurrent hypertensive disorders is associated with substantially higher risks of adverse perinatal outcomes [12]. This underscores the need for early booking in pregnancy for early identification and prompt management of problems.

Prevalence of PIH was found to be high thus the increase in the referrals due to PIH could be a reflection of the high prevalence of PIH. It could also be a reflection of the increase in booked pregnancies which is a consequence of the decrease in maternity fees. Individual factors such as obesity, multiple foetuses or mother’s ages were associated with high prevalence of PIH. A prospective study conducted by Bener and Saleh revealed that obesity increased the odds of developing PIH by 10 times [13]. While in our study we did not investigate the factors associated with PIH the rise in obesity among Zimbabwean women could explain the high prevalence. Obesity among women in Zimbabwe has increased from 1.2 % [14] in 2005 to 15.1 % in 2010 [15]. Other studies have also shown that obesity is a risk factor for PIH [16, 17] hence if such women were to become pregnant, they would be at higher risk of developing PIH.

Compared to the recommendations by the World Health Organisation (WHO), the caesarean section delivery rate of 12.5 % is above the recommended cut off. WHO recommends caesarean section rates between 5 % and 10 % and rates of 15 % are considered to do more harm than good. Literature suggests that cesarean section rates higher than the proposed 15 % upper threshold are associated with increased morbidity and mortality for both mothers and babies [18].

A population-based retrospective cohort study conducted in Zhejiang province in China in 1995-2000 demonstrates the importance of use of cesarean section during delivery among women with PIH. It was found that moderate and severe PIH early developed during pregnancy could increase the risk of perinatal mortality while the cesarean delivery could decrease the risks in women with PIH [19]. In our study, however, it was noted that among the reasons for caesarean section, some could have been avoided such as hands-to-belly movements to turn a breech. It was however also noted that providing continuous support to a woman during labour may be impossible due to the volume of deliveries compared to the number of midwives on any given shift.

A study by Rahman et al revealed that pregnancy-induced hypertension was an independent risk factor for low birth weight. Results from our study show that PIH was associated with delivering a low birth weight baby. Considering that low birth weight is a major determinant of mortality, morbidity and disability in infancy and childhood as well as long-term impact on health outcomes in adulthood, the increased risk due to PIH is a cause for concern. The costs of low birth weight on the health delivery system have also been documented [20] thus preventing and/or managing PIH becomes a priority as one of the ways of reducing the risk of low birth weight and the associated consequences.

According to the guidelines for the management of PIH in Zimbabwe, women with mild PIH should not receive any medication yet in this study more than half of the women received some form of medication. This could be a reflection of insufficient knowledge on the management of this condition as confirmed by the fact that less than half of the respondents could clearly articulate the definition and management of PIH. Hydralazine was given to one woman with moderate PIH and this was in line with the guidelines as she complained of one of the cardinal signs of imminent eclampsia – headache.

It was found in this study that urinalysis was only being done for those women with elevated blood pressure yet it is supposed to be routinely done for all pregnant women. This is an indicator of the scarcity of resources which results in the inability of maternity facilities to offer basic maternity services.

The scarcity of essential resources such as BP machines, ambulances and staff can contribute to the third delay in maternal and neonatal care thus increasing the risk of maternal and neonatal mortality. The third delay is the delay in receiving care, in this case, the delay in receiving care for a woman with PIH. This delay may be due to a variety of factors which include poorly trained or not sufficiently competent staff who lack understanding of the clinical relevance of PIH, poorly staffed health centres (brain drain, employment freeze), lack of drugs, equipment, and other supplies (tools of the trade), poor health care financing mechanisms as well as delays in referral to next level. Most of these factors were found to be present in our study thus this raises concern about the quality of management of pregnant women that is possible, particularly women requiring emergency care.

Results from our study also revealed that health workers from the central hospitals thought some of the referrals due to PIH were unnecessary yet discussions with key informants revealed that the primary level nurse did not take any chances if they thought the pregnancy was high risk. Thus, there is controversy over the indications for referral of women with PIH. Judging also from our results where we found that the standard protocol for management of PIH is unknown to the majority of the health workers, unnecessary referrals to tertiary hospitals cannot be ruled out.

Possible selection bias among women recruited into this study cannot be ruled out. The study was conducted over a short period of time thus we could have missed essential characteristics among women who did not get the chance to be part of this study.

## Conclusion

The prevalence of PIH was high. Women with PIH were at higher risk of adverse pregnancy outcomes than those without. Poor knowledge of management of PIH and inadequate resources are a threat to the proper management of PIH. This underscores the need for human resource capacity building and resource mobilisation for proper management of women accessing maternity services in Harare. Resources for routine urinalysis must be made available by hospital authorities.

### Study limitations

The study was a cross-sectional study conducted in half of the public maternity facilities in Harare. Possible selection bias can therefore not be ruled out. Those that were left out may have characteristics different from those that were enrolled into the study.

## Abbreviations

ANC: Ante-Natal Clinic
BP: Blood Pressure
PIH: Pregnancy Induced Hypertension
OR: Odds Ratio
WHO: World Health Organization

## References

[1] Palacios C and Pena-Rosas JP. Calcium supplementation during pregnancy for preventing hypertensive disorders and related problems. WHO RHL Commmentary. http://apps.who.int/rhl/pregnancy_childbirth/antenatal_care/nutrition/cd001059_penasrosasjp_com/en/**.** Accessed 19/11/2014

[2] Arshad A, Pasha W, Khattak T. A and Kiyani RB. Impact of Pregnancy Induced Hypertension on Birth Weight of Newborn at Term. Journal of Rawalpindi Medical College (JRMC);2011;15(2):113-115. Available at http://www.journalrmc.com/volumes/1394781698.pdf. accessed 19/11/2014

[3] Sibai, Baha.M. Diagnosis and Management of Gestational Hypertension and Preeclampsia. Obstetrics and Gynecology 2003 July; 102(1): Available from http://journals./www.com/greenjournal 10.1016/s0029-7844(03)00475-7 12850627

[4] Srinivas SK, Edlow AG, Neff PM, Sammel MD, Andrela CM and Elovitz MA. Rethinking IUGR in preeclampsia: dependent or independent of maternal hypertension? Journal of Perinatology. 2009;29:680–684. doi:10.1038/jp.2009.83; published online 16 July 2009 10.1038/jp.2009.83 PMC2834367 19609308

[5] Shweta Anand, *Kirshnanand, 2011. Perinatal Outcome in Growth Retarted Babies Born to Normotensive and Hypertensive Mothers: A Prospective Study. Available at http://www.pjsr.org/Jan12_pdf/5.%20Shewta%20Anand%20Dr..pdf. Accessed 19/11/2014

[6] Zhang J, Zeisler J, Hatch MC, Berkowitz G. Epidemiology of Pregnancy Induced Hypertension. Epidemiologic Reviews Vol 19, No. 2 (1997). Available at http://epirev.oxfordjournals.org/ [accessed on May 17, 2012] 10.1093/oxfordjournals.epirev.a017954 9494784

[7] Ministry of Health and Child Welfare (2010). National Child Survival Strategy for Zimbabwe 2010-2015. Harare, Zimbabwe. p12-26

[8] Ministry of Health and Child Welfare Zimbabwe (2007). Maternal and Perinatal Mortality Study. Harare, Zimbabwe. p17-24

[9] Tachiweyika Emmanuel, Gombe Notion, Shambira Gerald, Chadambuka Addmore, Tshimamga Mufuta, Zizhou Simukai ;Determinants of perinatal mortality in Marondera district, Mashonaland East Province of Zimbabwe, 2009: a case control study. Pan African Medical Journal. 2011 8:7. Available at http://www.panafrican-med-journal.com/content/article/8/7/full/. [accessed 18/05/2011] 10.4314/pamj.v8i1.71054 PMC3201615 22121416

[10] Hauth JC, Ewell MG, Levine RJ, Esterlitz JR, Sibai B, Curet LB et al. Pregnancy Outcomes in Healthy Nulliparas Who Developed Hypertension. Obstetrics & Gynecology 2000;95(1):24-28. Available at http://journals.lww.com/greenjournal/Abstract/2000/01000/Pregnancy_Outcomes_in_Healthy_Nulliparas_Who.5.aspx. [accessed 17/05/2012] 10.1016/s0029-7844(99)00462-7 10636496

[11] Latifah A. Rahman, Noran N. Hairi and Nooriah Salleh. Association Between Pregnancy Induced Hypertension and Low Birth Weight; A Population Based Case-Control Study. Asia Pac J Public Health. 2008;20(2):152–8. doi:10.1177/1010539507311553. 10.1177/1010539507311553 19124309

[12] Epidemiology. 2010 Impact of Pregnancy-Induced Hypertension on Stillbirth and Neonatal Mortality in First and Higher Order Births: A Population-Based Study: 21(1): 118–123. doi:10.1097/EDE.0b013e3181c297af. [Accessed 27 November 2012] 10.1097/EDE.0b013e3181c297af PMC2841017 20010214

[13] Bener A, Saleh NM (2013). The impact of socioeconomic, lifestyle habits and obesity in developing of pregnancy induced hypertension in fast growing country: Global comparisons. Clin Exp Obstet Gynaecol.

[14] Ministry of Health & Child Welfare, 2005. National Survey Zimbabwe Non-Communicable Disease Risk Factors - (ZiNCoDs) Preliminary Report. Harare, Zimbabwe. p65,66

[15] Zimbabwe National Statistics Agency (ZIMSTAT) and ICF International (2012). Zimbabwe Demographic and Health Survey 2010-11.

[16] Robinson HE, O'Connell CM, Joseph KS, McLeod NL; Outcomes in Pregnancies Complicated by Obesity. Obstet Gynecol. 2005;106:1357–64 [accessed 27 May 2013]. 10.1097/01.AOG.0000188387.88032.41 16319263

[17] Shabnam S and Humaira N. Pregnancy with Obesity -A Risk Factor for PIH. JLUMHS SEPTEMBER-DECEMBER 2010; Vol: 09 No. 03. [accessed 27 May 2013]

[18] Chu K, Cortier H, Maldonado F, Mashant T, Ford N, Trelles M; Cesarean Section Rates and Indications in Sub- Saharan Africa: A Multi-Country Study from Medec ins sans Fronti eres. PLoS ONE. 2012;7(9), e44484. doi:10.1371/journal.pone.0044484 [accessed 27 November 2012]. 10.1371/journal.pone.0044484 PMC3433452 22962616

[19] Ye RW, Liu YH, Ma R, Ren AG, Liu JM; Association between pregnancy-induced hypertension, cesarean delivery and perinatal mortality: a prospective study. Zhonghua Liu Xing Bing Xue Za Zhi. 2009 Sep;30(9):891-4. Available at http://www.ncbi.nlm.nih.gov/pubmed/20193221 [accessed 28/05/2012] 20193221

[20] WHO. Feto-maternal nutrition and low birth weight. Available at www.who.int/nutrition/…en/index.html. [Accessed 29 November 2012]

